# Estimating the direct costs of ischemic heart disease: evidence from a teaching hospital in BRAZIL, a retrospective cohort study

**DOI:** 10.1186/s12872-017-0615-1

**Published:** 2017-07-04

**Authors:** Rosane Paixão Schlatter, Vânia Naomi Hirakata, Carisi Anne Polanczyk

**Affiliations:** 1 Graduate Cardiology Program, Universidade Federal do Rio Grande do Sul (UFRGS), Porto Alegre, RS Brazil; 2 Department of Cardiology, Hospital de Clinicas de Porto Alegre (HCPA), Porto Alegre, RS Brazil; 3 Department of Cardiology, School of Medicine, UFRGS, Porto Alegre, RS Brazil; 4 National Health Technology Assessment Institute, CNPq, Porto Alegre, RS Brazil; 5 Research and Graduate Studies Department. Hospital de Clinicas de Porto Alegre HCPA, Porto Alegre, RS Brazil; 6 Programa de Pós-graduação em Cardiologia, Hospital de Clínicas de Porto Alegre, Rua Ramiro Barcelos, 2350, CEP, Porto Alegre, RS 90035-903 Brazil

**Keywords:** Direct costs, Coronary artery disease, Disease cost study

## Abstract

**Background:**

Coronary artery disease is the most prevalent cardiovascular disease. In the United States, 7% of adults over 20 years of age are estimated to have coronary artery disease. In Brazil, a prevalence of 5 to 8% has been estimated in adults over 40 years of age, with an increased number of hospitalizations associated with both stable and acute clinical manifestations; and health care costs have quadrupled in the last decade. To estimate the direct costs of managing ischemic heart disease patient care in a teaching hospital in Brazil from the perspective of the service payer, the Brazilian Unified Health System.

**Methods:**

This study was a retrospective cohort study for the identification and valuation of resources used at both the outpatient and in-hospital levels in a sample of 330 patients selected from the hospital's ischemic heart disease clinic. Data were collected from computerized hospital records and patients' hospital bills from January 2000 to October 2015. A bivariate analysis and binary logistic regression were performed with p < 0.05 considered statistically significant.

**Results:**

The study population consisted of 330 patients with a mean age 61 ± 10 years and a follow-up period of 107 ± 2.6 months; of the patients, 55% were male, 89% had hypertension, 48% had diabetes, and 65% had acute myocardial infarction. The mean annual cost of outpatient management was US $1,521 per patient. The mean cost per hospitalization was US $1,976, and the expenses were higher in the first and last years of follow-up. Unstable angina, revascularization procedures, diabetes, hypertension and obesity were predictors of higher hospitalization costs (p <0.05).

**Conclusion:**

The cost estimates in this study indicate a high proportion of drug treatment costs in the treatment of ischemic heart disease. Treatment costs are higher in the first year and at the end of treatment, and some clinical factors are associated with greater hospital care costs. These results may serve as a basis for the evaluation of existing public policies and inputs for cost-effectiveness studies in coronary artery disease.

**Trial registration:**

CEP HCPA 11–0460. Ethics Committee of Hospital de Clínicas de Porto Alegre.

## Background

In 2012, cardiovascular disease accounted for 46.2% of non-communicable chronic disease deaths and 37% of deaths in people aged under 70 worldwide [1]. In Brazil, despite a reduction in the number of deaths since the 1990s, cardiovascular disease accounted for 28% of deaths in 2013 and incurred the highest spending in hospitalization in the Brazilian Unified Health System (SUS) in 2009 [2–4].

Coronary artery disease (CAD) is the most prevalent cardiovascular disease. In the United States, 7% of adults over 20 years of age are estimated to have a coronary artery disease diagnosis; by 2030, there will be an increase in this indicator [5]. In Brazil, a prevalence of 5 to 8% has been estimated in adults over 40 years of age [6], with an increased number of hospitalizations associated with both stable and acute clinical manifestations [6, 7].

Health costs have been increasing since the 1980s, mainly due to the incorporation of new technologies and demographic and epidemiological population transitions. In Brazil, health care costs have quadrupled in the last decade, reaching US $125 billion in 2013, of which 44% was paid by public health and 56% by the private sector [8].

The increase in spending on health, the need to balance public budgets and to seek efficiency in the allocation of resources, and the requirement of taking into account social demands in their entirety has led to a growing number of economic studies to aid in the decision-making process regarding the implementation of new public health policies.

Disease cost studies are important in Brazil because they provide a baseline for complete economic evaluations, such as cost-effectiveness studies. Identifying costs involves proper planning for data collection to enable the use of the generated information as an aid for future studies. In this context, this study aimed to estimate the direct costs associated with the treatment of ischemic heart disease at the outpatient and in-hospital levels in a public teaching hospital from the perspective of the main service public payer in Brazil, SUS.

## Methods

This study was a retrospective cohort study to identify and value resource use in ischemic heart disease outpatients of the Hospital de Clínicas de Porto Alegre between January 2000 and October 2015. We used a bottom up microcosting methodology for 330 patients estimating direct costs during more than 10 years follow up. The inclusion criteria in this study were outpatients with follow-up of more than or equal to one year and a minimum of 3 consultations since January 2009. Of the 633 patients followed up in the ischemic heart disease clinic, 330 met the inclusion criteria and were included in the study.

### Data collection

Forms were developed using REDCap [9] to collect retrospective data related to ambulatory care protocol clinical information. The information included CAD risk factors, medical history and current physical assessment, medication used, hospitalizations, procedures and tests performed during follow-up. Resource use for outpatient treatment and for hospitalization were analyzed separately.

Information relative to the following factors were recorded on the clinical assessment form: (A) Canadian Cardiovascular Society (CCS) functional class, (b) previous diagnosis of unstable angina, myocardial infarction, stroke, and congestive and valvular heart failure, (c) risk factors (diabetes mellitus, hypertension, dyslipidemia, and current and previous smoking), (d) medication use and (e) physical examination (weight, height, systolic and diastolic blood pressure, and heart rate).

### Quantification of resources

The following factors were quantified during outpatient treatment: medical appointments, tests, outpatient catheterization procedures, medications in use, and transport of the patient to the hospital. Only medical consultations that took place in the ischemic heart disease clinic were considered. The number of consultations performed per patient was recorded.

The following drugs in use were considered in this study: acetylsalicylic acid, allopurinol, amiodarone, amlodipine, atenolol, atorvastatin, benazepril, captopril, clopidogrel, digoxin, diltiazem, enalapril, furosemide, hydrochlorothiazide, glibenclamide Isosorbide, losartan, lovastatin, metformin, metoprolol, nifedipine, omeprazole, paracetamol, pravastatin, propranolol, simvastatin, sustrate, warfarin, and verapamil. The list of drugs that were considered in this analysis is not exhaustive. The drugs included in the study were the ones prescribed by the cardiologists who tended to the patients while the study was ongoing as well as hypoglycemic drugs. All drugs were recorded in mg/day. We assumed that the drugs were purchased by the patients at pharmacies using their own resources without government subsidies and were taken with 100% treatment adherence.

The laboratory tests included alanine aminotransferase (ALT), albuminuria, aspartate aminotransferase (AST), total calcium, creatine kinase (CK and CK-MB), high density lipoprotein (HDL) cholesterol, low density lipoprotein (LDL) cholesterol, total cholesterol, creatinine, glycemic curve, qualitative urine test (QUT), urinary sediment test, phosphorus, glucose, glycated hemoglobin, potassium, C-reactive protein, thyrotropin, triglycerides, and urea.

Cardiac and imaging tests included were myocardial scintigraphy (stress and resting), echocardiography, stress echocardiography, resting electrocardiography, exercise test, Holter, ambulatory blood pressure monitoring (ABPM), chest X-ray, carotid and vertebral Doppler echocardiography, venous color Doppler echocardiography, and chest tomography.

The following factors were quantified in terms of hospital treatment: hospitalization days, intensive care unit (ICU) days, laboratory and diagnostic tests, angioplasty procedures with or without stent implantation, cardiac catheterization, and coronary artery bypass surgery performed during hospitalization.

Three pieces of information were evaluated for the hospitalizations (disease code, treatment code charged to the hospital account, and the specialty responsible for the hospitalization). The study included hospitalizations with International Classification of Diseases and Related Health Problems (ICD-10) relating to Chapter IX - Diseases of the Circulatory System (I00 to I99), Chapter XVIII - Symptoms, signs and abnormal clinical and laboratory findings, not classified elsewhere (R00, R07–4, R53, and R55), Septicemia, unspecified (A41.9), unspecified Diabetes Mellitus (E14), Transient cerebral ischemic attacks and related syndromes (G45), Prosthetic device, implant and cardiac and vascular graft complications (T82.0 to T82.9), Fitting and adjustment of other external prosthetic devices (Z44.8) and presence of implants and cardiac and vascular grafts (Z95.0 to Z95.9).

Death, hospitalization and coronary artery bypass surgery were defined as major events.

#### Resource valorization

The study used the SUS reimbursement system to estimate costs. The costs of consultations and tests were calculated by multiplying the quantity by the unit price of each resource used in the SUS table.

For pharmacological treatment, a survey of drug prices was performed in the same physical form across three pharmaceutical networks, and their mean prices were calculated. The average price of the medicines was calculated based on the generic version of the drug whenever it was available in Brazil. From the list of medications considered, only 2 drugs do not have the generic version available in Brazil: benazepril by the Novartis Laboratory and sublingual isosorbide-5 mononitrate by the Baldacci Laboratory. For patient transport costs, the price of public land transport in force in October of 2015 was used by assuming 2 journeys for residents in Porto Alegre and 4 journeys for residents in the metropolitan area, in the state interior, and in other states.

For hospitalization costs, the hospital bill amounts paid were used and adjusted for inflation by the National Consumer Price Index (Índice Nacional de Preços Amplo - IPCA) [10] considering the month of closure of the hospital bill as the initial date and October 2015 as the final date.

The total cost of each patient included outpatient care and hospitalization and was divided by the follow-up period to obtain the mean annual cost per individual. The costs were expressed as median values ​​in Brazilian currency and converted to US dollars at an exchange rate of 1 Real = US $3.60 (exchange rate of 10/2016). For the estimated annual hospitalization cost per patient, analyses were performed to identify potential clinical predictors of higher cost considering the following variables: gender, age, CCS functional class, stroke, unstable angina, myocardial revascularization surgery, myocardial infarction, diabetes mellitus, dyslipidemia, hypertension, body mass index, and active and prior smoking status.

#### Statistical analysis

Descriptive data are presented as absolute and relative frequencies for categorical variables and as means and standard deviations or medians and interquartile ranges (25th and 75th percentiles) for continuous variables. The Kolmogorov-Smirnov test was used to evaluate the data normality. The costs are presented as the mean and median. The cost variables were compared in relation to clinical predictors using the Mann-Whitney test for data with non-normal distributions after logarithmic transformation (bivariate analysis). A generalized linear model analysis (GLM) was performed to evaluate independent predictors of annual cost per patient. Binary logistic regression was performed to estimate the probabilities of major events and to establish a relationship with the estimated costs. A value of *p* < 0.05 was interpreted as statistically significant. To estimate the score, the cost ratio for significant risk factors was used and the individual probabilities of the presence of major events were calculated.

## Results

A total of 633 patients were evaluated, of whom 330 met the inclusion criteria and constitute the study population. A total of 54.8% were male, and 51.8% lived in Porto Alegre. The mean age was 61 years, and the mean follow-up period in the ischemic heart disease clinic was 107.38 ± 2.62 months. Patients with CCS functional class I accounted for 73% of the sample, and patients with CCS class II accounted for 20.6%. There were 31 deaths (9.4%) during the follow-up period, of which 5 were related to concomitant malignancies. The baseline characteristics of the sample are presented in Table 1.

**Table 1:** Basic population characteristics (*n* = 330)

Characteristics	N (%)
Male gender	181 (54.8)
Mean age (years)*	61 ± 10.3
Acute myocardial infarction	204 (61.8)
Unstable angina	94 (28.5)
Coronary artery bypass surgery	102 (30.9)
Stroke	21 (6.4)
Heart failure	18 (5.5)
Angioplasty	169 (51.2)
Diabetes mellitus	115 (34.8)
Arterial hypertension	277 (83.9)
Dyslipidemia	185 (56.1)
Current smoking	53 (16.1)
Previous smoking	196 (59.4)
Obesity	86 (26.1)
Overweight	139 (42.1)
Regular physical activity	109 (33.0)
CCS class	
I	241 (73.0)
II	68 (20.6)
III	17 (5.2)
IV	4 (1.2)
Patient origin	
Porto Alegre	171 (51.8)
Metropolitan region	122 (37)
Interior	35 (10.6)
Other states	2 (0.6)
SBP*	138.7 (80–230)
DBP*	82.6 (50–160)
Follow-up time**	107.38 ± 2.62

*expressed as the mean and interquartile range. **expressed in months CCS = Canadian functional class; SBP = Systolic blood pressure; DBP = Diastolic blood pressure

The use of outpatient resources in the follow-up period consisted of 9264 consultations, 71,114 laboratory tests, 5697 diagnostic tests and 416 outpatient catheterizations. On average, there were 28.1 consultations (median 28 and standard deviation 13.11) and 2 catheterizations (median 2 and standard deviation 1.44) per patient. The patients’ travel costs based on a return journey from their residence to the hospital totaled US $47,955 during the follow up. On average, the patients used 6 drugs per day, representing a mean cost of US $6174 (median US $5519) and a total of $2,037,298 during the period. Drug costs are shown in Table 2.

**Table 2:** Unit prices from the SUS table

Resources	US$ price
Consultations	2.10
Hospitalization days*	NA
ICU III days	141.29
Procedures**	
Coronary Angioplasty	712.25
Primary Coronary Angioplasty (including catheterization)	1303.28
Coronary Angioplasty with stent implantation	1277.39
Coronary Angioplasty with two stents implantation	2090.00
Cardiac catheterization	365.84
Myocardial Revascularization with extracorporeal use	3679.46
Diagnostic tests	
Myocardial scintigraphy with dipiridamol	113.48
Stress myocardial scintigraphy	113.48
Resting myocardial scintigraphy	106.41
Echocardiogram	11.09
Stress echocardiogram	45.83
Electrocardiogram	1.43
Exercise test	8.33
Holter	8.33
Ambulatory Blood Pressure Monitoring	2.80
BP Chest X-ray	1.81
Venous color Doppler ultrasound	11.00
Laboratory tests	
Calcium	0.51
CK	1.02
CK-MB	1.14
HDL and LDL cholesterol	0.98
Total cholesterol	0.51
Creatinine	0.51
Glycemic curve	1.01
Qualitative urinary test	1.03
Phosphorus	0.51
Glucose	0.51
Glycosylated hemoglobin	2.17
Potassium	0.51
C Reactive protein	0.79
Glutamic-oxaloacetic transaminase (GOT)	0.56
Glutamic-pyruvic transaminase (GPT)	0.56
Thyrotropin	2.49
Triglycerides	0.98
Troponin	2.50
Urea	0.51
Generic Drugs***	
Acetylsalicylic acid 100 mg	0.06
Allopurinol 100 mg	0.08
Amiodarone 200 mg	0.26
Amlodipine 5 mg	0.37
Atenolol 50 mg	0.22
Atorvastatin 20 mg	0.73
Captopril 12.5 mg	0.16
Captopril 25 mg	0.22
Captopril 50 mg	0.46
Clopidogrel 70 mg	1.45
Digoxin 0.25 mg	0.10
Diltiazem 60 mg	0.16
Enalapril 5 mg	0.24
Enalapril 10 mg	0.21
Enalapril 20 mg	0.36
Furosemide 40 mg	0.12
Hydrochlorothiazide 25 mg	0.20
Glibenclamide 5 mg	0.08
Isosorbide 10 mg	0.08
Losartan 50 mg	0.34
Lovastatin 20 mg	0.41
Metformin 850 mg	0.23
Metoprolol 100 mg	0.19
Nifedipine 10 mg	0.25
Omeprazole 20 mg	0.25
Paracetamol 500 mg	0.16
Pravastatin 10 mg	0.44
Propranolol 40 mg	0.06
Propranolol 80 mg	0.09
Simvastatin 10 mg	0.63
Simvastatin 40 mg	0.51
Sustrate 10 mg	0.13
Warfarin 5 mg	0.10
Verapamil 80 mg	0.20
Non-generic drugs***	
Benazepril 5 mg	0.54
Sublingual Isosorbide-5 mononitrate	0.11

*Inpatient stay in the SUS are included in the payment for the treatment performed. ** Includes normatives income. *** price for one tablet

The total outpatient costs during the follow-up period were US $2,432,933 (Table 3), with a mean annual cost per patient of $854 (median US $765). The greatest drivers of the outpatient costs were pharmacological treatment (81%) and imaging tests (7%).

**Table 3:** Outpatient costs during the follow-up period in US$

	Consultations	Laboratory tests	Diagnostic tests	Drugs	Transport	Catheterization	Total outpatient cost
N	330	326	325	330	330	208	330
Mean	58.88	178.92	497.91	6.173.63	145.32	519.73	7372.53
Median	58.72	149.96	407.31	5.518.92	74.03	341.51	6539.51
Minimum	6.29	10.58	7.15	0.00	7.22	170.76	275.32
Maximum	159.39	913.76	1742.44	23,560.92	4613.83	2788.11	26,193.39
Total	19,428.67	58,328.71	161,819.60	2,037,297.67	47,954.91	108,104.27	243,293.83

The mean estimated total hospitalization cost during the follow-up period was US $6104 (median US $4086), which generated a mean cost per hospitalization of US $1976 (median US $1710) (Table 4).

**Table 4:** Hospitalization costs*

Mean cost per hospitalization	US$ value
N	251
Mean	1976.21
Median	1710.39
Standard deviation	1231.69
Minimum	103.82
Maximum	11,014.21
Cumulative cost of hospitalizations	
Mean	6104.76
Median	4086.02
Standard deviation	5843.72
Minimum	103.82
Maximum	38,890.29
Total	1,532,293.84
Costs per patient considering the outpatient and hospitalization basis	
Mean	12,015.84
Median	9726.93
Standard deviation	8134.67
Minimum	275.32
Maximum	59,437.76
Annual cost per patient	
Mean	1521.54
Median	1217.10
Standard deviation	1171.49
Minimum	237.79
Maximum	8171.83

*Includes tests, procedures, specialist materials, ICU days and fees when incurred

Regarding the total in-hospital procedures performed, catheterizations accounted for 33% (*n* = 186), angioplasties for 53% (*n* = 294) and coronary artery bypass surgery for 12% (*n* = 66). A total of 102 laboratory tests (median 52) and 11 imaging tests (median 7) were performed on average for each patient hospitalized during the follow-up period. In total, 26,092 laboratory tests and 2484 imaging tests were performed during the follow-up period.

**Fig. 1:**
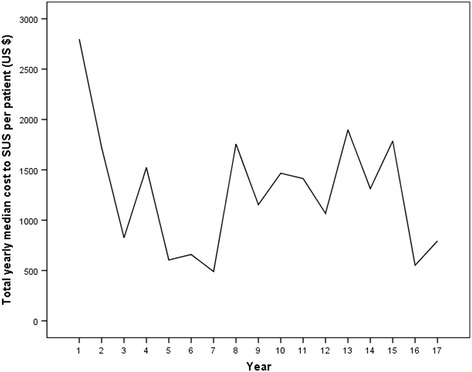
Total cost to the SUS per year of follow-up (median)

Analysis of the total cost for the SUS showed that the highest costs occurred during the first year of follow-up; these costs average US $2865 per hospitalization, with a median of US $2799. The highest number of admissions also occurred during this period (Fig. 1).

**Fig. 2:**
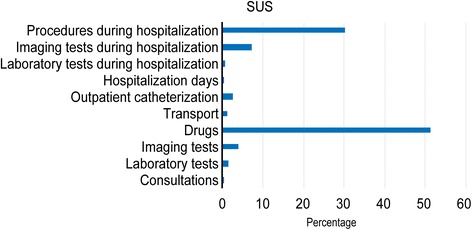
Individual costs as a proportion of the total cost during follow-up

Considering outpatient and hospitalization costs as a whole, the estimated total costs were US $3,965,228, with a mean annual cost per patient of US $1522 and median of US $1217 (Table 4). In this study, hospital costs accounted for 38.6% of the total cost of managing ischemic heart disease and outpatient costs accounted for 61.4%. The largest driver of costs was expenditure on drugs, followed by procedures during hospitalization (Fig. 2).

Significant factors that drove the greatest annual costs per patient in hospitalization included unstable angina, angioplasty, prior infarction bypass surgery, diabetes mellitus, hypertension, and obesity. Male gender and age were associated with greater hospitalization costs, but this result was not maintained after the multivariate adjustment (GLM) (Table 5).

**Table 5:** Clinical predictors of the annual cost per patient

	Male			Female		P*
	Mean	Median		Mean	Median	
Annual cost per patient	1556.14	1171.74		1454.52	1298.85	0.884
	≤74 years			≥ 75 years		
	Mean	Median		Mean	Median	
Annual cost per patient	1508.29	1212.88		1649.38	1272.95	0.809
	Stroke			No Stroke		
	Mean	Median		Mean	Median	
Annual cost per patient	1727.86	1301.13		1507.52	1199.09	0.158
	Unstable angina			No Unstable angina		
	Mean	Median		Mean	Median	
Annual cost per patient	1742.73	1490.25		1433.45	1119.83	0.002
	CABS			No CABS		
	Mean	Median		Mean	Median	
Annual cost per patient	1649.55	1422.75		1464.28	1156.82	0.01
	AMI			No AMI		
	Mean	Median		Mean	Median	
Annual cost per patient	1578.52	1155.48		1429.29	1260.28	0.562
	Diabetes			No Diabetes		
	Mean	Median		Mean	Median	
Annual cost per patient	1781.96	1500.19		1382.25	1144.14	< 0.01
	Dyslipidemia			No Dyslipidemia		
	Mean	Median		Mean	Median	
Annual cost per patient	1475.45	1158.71		1580.36	1265.95	0.271
	Hypertension			No Hypertension		
	Mean	Median		Mean	Median	
Annual cost per patient	1568.30	1232.89		1277.17	1183.72	0.03
	BMI ≤ 29.9			BMI ≥ 30		
	Mean	Median		Mean	Median	
Annual cost per patient	1451.13	1145.89		1629.54	1498.74	0.011
	Current smoking			No Current smoking		
	Mean	Median		Mean	Median	
Annual cost per patient	1398.59	1059.38		1558.75	1179.43	0.838
	Previous smoking			No Previous smoking		
	Mean	Median		Mean	Median	
Annual cost per patient	1678.31	1059.38		1523.99	1177.12	0.545
	Heart failure			No Heart failure		
	Mean	Median		Mean	Median	
Annual cost per patient	2323.57	1.482.99		1475.28	1209.04	0.179
	Angioplasty			No Angioplasty		
	Mean	Median		Mean	Median	
Annual cost per patient	1832.63	1491.66		1195.00	976.29	< 0.01

*Mann-Whitney test

**Fig. 3:**
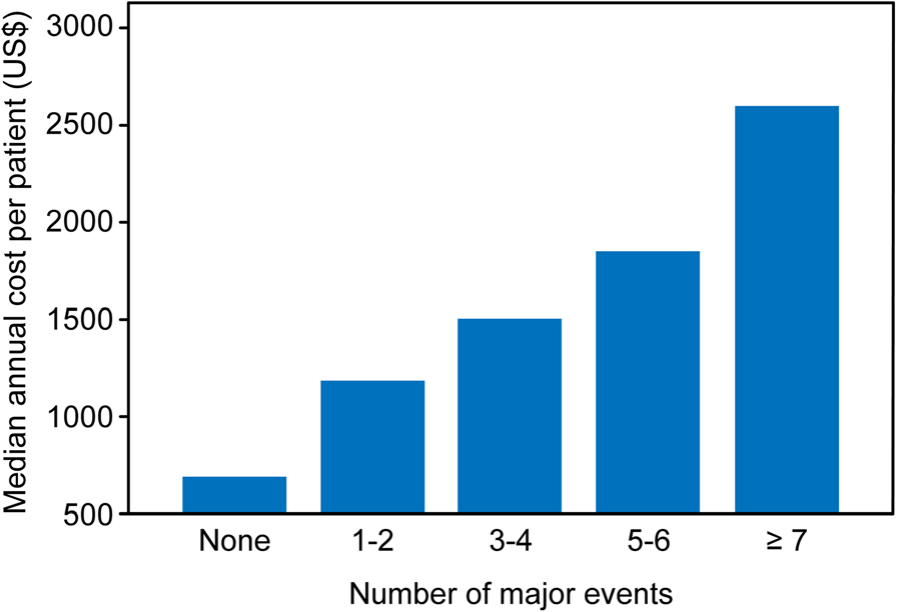
Mean annual cost per patient considering the number of major events

**Fig. 4:**
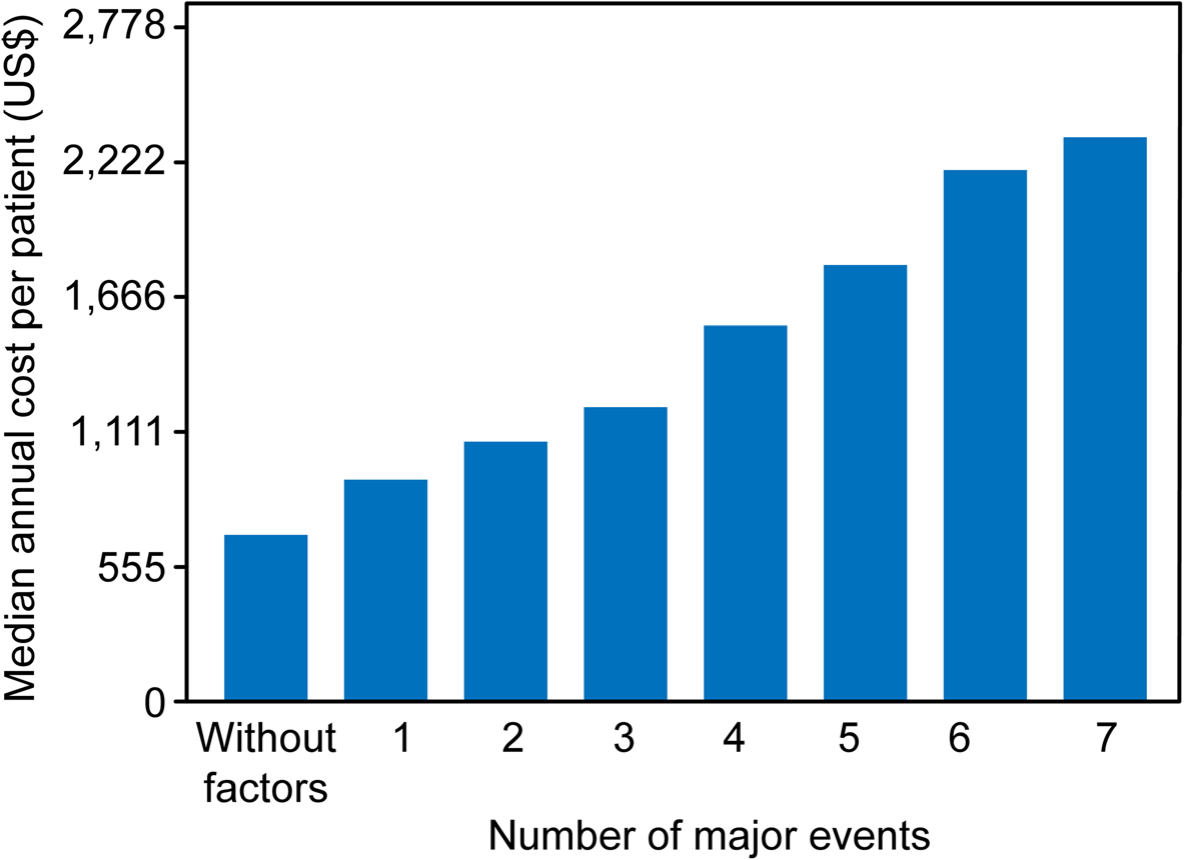
Median annual cost per patient according to significant clinical predictors

Because this study involved a specialized outpatient clinic and a cohort with 10 years of follow-up, 76.7% of the patients had an event during this period. The estimated total cost for the SUS was assessed in relation to the likelihood of major events (hospitalization, death, and coronary artery bypass surgery) and the estimated annual cost per patient by considering the presence of significant risk factors in the bivariate analysis. The results are shown in Figs. 3 and 4.

## Discussion

The results of this study offer up-to-date data regarding the outpatient and hospital management cost of patients undergoing clinical follow-up in an ischemic heart disease clinic in a public teaching hospital.

When estimating costs, some economic factors must be considered in the analysis, such as market prices, inflation, depreciation of goods, and opportunity cost [11]. Because this study involved a cohort with a ten-year follow-up, the amounts received in the years prior to the analysis were adjusted for inflation. The price tables used by the SUS were used to estimate the costs, with the exception of drug prices. The prices used by the SUS do not reflect the true costs of the intervention, rather reimbursement values, but represent the closest ones. They can be considered the opportunity cost of each system, which is considered by some authors to represent the real economic value of a resource used to perform an intervention [12, 13]. For the drugs price, we used the average price of the drugs; the estimates would be more accurate if it were possible to identify the amount paid by the government for the medication. However, in the Brazilian government’s health database, called DATASUS, this information is not available.

In Brazil cost management accounting in the health area is a rather recent theme and institutions are still developing initiatives to know the real costs of patient care. In our hospital, there is no cost management system at the moment and thus, we cannot accurately state what percentage of the costs we find represents the real costs incurred. Since 2008, the readjustments of the Brazilian Health System reimbursements do not reflect Brazilian inflationary indices such as the National Broad Consumer Price Index (IPCA) and the National Consumer Price Index (NCPI). According to the Brazilian Federal Council of Medicine, in the performance of a coronary angioplasty with stent implantation this gap varies between 39% for the IPCA and 40% for the INPC.

Although previous studies are scarce in the Brazilian literature, some have described the management costs of cardiovascular disease patients. Below, some of these studies are discussed, and the results prior to 2015 have been updated for inflation and are presented in this format for comparisons.

The mean annual cost per outpatient in the SUS was US $854 (median US $765); this finding was similar to the result reported by Ribeiro et al. [7] in a study conducted in 2002, which reported an annual per patient expenditure of US $779 (median US $760) with the same cohort of patients, and by Azambuja et al. [14], who estimated a mean annual cost of US $875 for cases of severe cardiovascular disease.

In terms of the composition of outpatient costs, spending on drug treatment was responsible for the highest expenditure. Regarding the mean annual cost of drugs, the studied sample showed values ​​similar to US $830 found by Araújo et al. in heart failure patients [15]. This expenditure as a proportion of the total outpatient cost (81%) was higher than that found by Ribeiro et al. [7] (80%). This finding is consistent with the reality of Brazilian health care and may reflect the entry of new drugs with higher prices into the market, an increasing number of concomitant cardiovascular diseases, and cumulative price inflation. The cost results presented are similar to those of 10 years ago and we consider that this similarity can be partly explained by the gap in the correction of the Brazilian Health System reimbursements. For example, the value of the medical consultation for reimbursement is US $2.10 since 2005; similarly, several laboratory tests requested in the routine care of patients with ischemic heart disease remain unchanged at US $0.51. Another example, the value of stress scintigraphy found by Ribeiro was US $93.33; using by the IPCA index, it should be equivalent to US $167.63 (a variation of 79.61%) in this study. However, the reimbursement system only pays US $113.48 (a variation of 21.59%). This study considered that patients paid for 100% of the drugs and that there was 100% drug adherence, which might have led to overestimation of the total costs. Indeed, hypertension, diabetes, and dyslipidemia drugs are provided free in Primary Health Care Units (PHCUs) to public health system users, and there are government subsidies when these drugs are purchased by the private health system users as part of the National Pharmaceutical Assistance Policy [16]. Regardless, patient accessibility to this program was not ascertained in this study. In relation to treatment adherence in Brazil, Tavares et al. [17] found a high rate of adherence used to treat high blood pressure, ranging from 71% in the North to 84% in the South. Santos-Pinto et al. [18] analyzed the Popular Pharmacy Program aimed at private system users and estimated that approximately 70% of users in the North and Northeast who could obtain the drugs for free in the PHCUs acquired them from the Popular Pharmacy Program due to access problems in the PHCUs.

The mean cost to the SUS of each hospitalized patient of US $1976 was similar to the amount reported by Teich [4] (US $2005) in a study of acute coronary syndrome.

The analysis of the total cost to the SUS for each patient during the period suggests that the total costs were higher in the first year and at the end of follow-up, but it was not possible to establish an expenditure distribution pattern. When considering the major events in this cohort (*n* = 1021), the clinical variables that were predictive of increased costs were unstable angina, angioplasty, coronary artery bypass surgery, diabetes mellitus, obesity, and hypertension, with hospitalizations and readmissions responsible for 93% of all events. The higher number of hospitalizations during follow-up is consistent with literature, which shows re-hospitalization rates of over 30% during the first year of follow-up [16, 19]. As expected, the greater probability of events was associated with higher costs, which underscores the finding that the main drivers in this regard are related to the presence of severity markers, and consequent instability and need for hospitalization.

In this study, the outpatient costs were proportionately higher than the hospitalization costs in relation to the total cost primarily due to the drug expenditure. This result is different from the results found in the literature. The study with one year of follow-up conducted by Araujo [15] reported costs of 40% for hospitalization and 39% for medication. This difference may have been due to the data collection method used when quantifying drug use. In the study by Araújo, drug prices were calculated using the Brasíndice table in 2002. The Brasíndice table was a table published by a specialized company that had the price of drugs sold in Brazil, including the factory price and the maximum price to the consumer, plus taxes. Since May 2009, by resolution of the Regulation Chamber of the Brazilian Medicines Market, it is not allowed to divulge the maximum price of drugs to the consumer. We highlight that the Araújo study was carried out in 2002 with a cohort of 70 patients and used the methods of identifying the cost items and cost tables available at the time to estimate the actual costs of one year of treatment, including applying an overhead rate to the cost components. However, Araujo estimated the cost of hospitalization through the prospective analysis of 3 patients to identify the use of resources and establish the average cost of a day of hospital stay (top-down microcosting). Today the methods used by Araujo could be considered a mix of the bottom-up and the top-down microcosting approaches. The top-down approach usually produces higher values than the bottom-up approach. Our study used the bottom-up micro-costing approach for identifying and quantifying all cost components. We believe that the differences found may also be influenced by the different methodological approaches used. In the United States, Sieck [20] estimated hospital costs as being responsible for 60% and drugs for 10% of direct costs in patients with congestive heart failure. Dunlay et al. [21] reported that 77% of costs in heart failure were incurred during hospitalization. The difference between our results and those of international studies may be that the hospitalization figures do not account for Brazilian inflation rates. Thus, the prices charged were below inflationary correction for the period. Another factor that may have contributed to this result was the reduction in the cost of orthotics and prostheses (stents) in recent years. In early 2000, the costs of these products were high, but the costs have subsequently reduced due to the entry of a wide range of products from the Indian and Chinese markets into the Brazilian market [22]. Moreover, in the past, stents were acquired by the hospitals with the insertion of a profit margin in the transfer to the hospital account but, currently, are acquired directly by the health plans, and the supplier is paid directly.

In the bivariate analysis, clinical factors such as heart failure, acute myocardial infarction, dyslipidemia, and stroke were not confirmed as major cost predictors. This finding confirmed the results of other studies [14, 23, 24]. Diabetes mellitus and unstable angina were the major cost drivers at both the outpatient and in-hospital levels. The association of the number of major events with costs can be explained by the long follow-up period of this cohort and the number of hospitalizations. A study of heart failure patients showed that 51.2% of the patients return to the emergency room between 1 and 12 times over a one-year period [25].

Considering the 2012 demographic census for the population over 18 years is 9,548,247 people and a prevalence of 7% for ischemic heart disease, the annual direct costs to the SUS for ischemic heart disease would be at least US $3.66 billion. This figure is similar to the figure reported by Teich [4].

Some limitations of this study should be mentioned. Costs related to drugs were assumed to be constant throughout the follow-up period, and the existence of subsidies from governmental pharmaceutical assistance programs and treatment adherence were not considered, which may have caused the share of these costs in the analysis to have been disproportionately high. Another limitation is that the costs in this study were calculated based on the value of the service provided and did not include institutional operating costs. This approach may have led to underestimation of the presented costs.

## Conclusion

This study showed that the direct costs of longitudinal ischemic heart disease treatment were high and were primarily driven by chronic pharmacological treatment and days of hospitalization; through out a 10-year period the costs were higher in the first year and the last years of follow-up.

In 2014, the per capita income of the Brazilian population was US $292. Thus, the cost of drugs in this study suggests the need to expand the National Pharmaceutical Assistance Policy to include drugs related to coronary heart disease and to review the drug distribution policy in the PHCUs to provide access to drugs and reduce morbidity and mortality.

Data from this study could help to define health care policies for ischemic heart disease patients in terms of the allocation of human resources (physicians and care staff), physical resources (admissions, tests, and drugs), and appropriate financial planning.

## Abbreviations

ABPM: Ambulatory blood pressure monitoring
ALT: Alanine aminotransferase
AST: Aspartate aminotransferase
CAD: Coronary artery disease
CCS: Canadian Cardiovascular Society
CK: Creatinine kinase
GLM: Generalized linear model
HDL: High density lipoprotein
ICD: International Classification of Diseases and related Health Problems
ICU: Intensive care unit
IPCA: National Broad Price Index
LDL: Low density lipoprotein
PHCUs: Primary Health Care Units
QUT: Qualitative urine test
REDCap: Research electronic data capture
SUS: Unified Health System
